# Determination of β-catenin Expression in Breast Cancer and Its Relationship with Clinicopathologic Parameters

**DOI:** 10.31557/APJCP.2021.22.11.3493

**Published:** 2021-11

**Authors:** Salma Sefidbakht, Hanieh Saeedipour, Hiva Saffar, Elham Mirzaian

**Affiliations:** *Department of Pathology, School of Medicine, Shariati Hospital, Tehran University of Medical Sciences, Tehran, Iran.*

**Keywords:** β-catenin, breast cancer, clinicopathologic parameters

## Abstract

**Background:**

Abnormal activation of the β-catenin signaling pathway is involved in various malignancies, including breast carcinoma.Aberrant expression of β-catenin has been associated with more aggressive behaviors of breast cancer in some previous studies. . In the present study, we intend to evaluate the β-catenin expression in breast cancer specimens and study its relationship with clinicopathological parameters.

**Materials and method:**

In this cross-sectional study,88 samples diagnosed as invasive ductal breast carcinoma from 2007 to 2017 were evaluated. The slides and paraffin blocks were retrieved from the archive of pathology department. Patients’ clinical characteristics and other information were also extracted from medical documents. Sections from related paraffin blocks through the tissue microarray method were provided, and immunohistochemistry staining for β-catenin was done. Then different patterns of β-catenin expression and the relationship between different patterns and clinicopathological parameters were investigated.

**Results:**

Of the 88 breast cancer samples, 94% were female, and 6% were male. In 70% of the samples, normal membrane expression of β-catenin was observed. Whereas in 30% of them, aberrant expression of β-catenin was observed. A close significant relationship was observed between aberrant β-catenin expression and age over 50 years (p-value: 0.093) and negative HER2 (p-value: 0.07).

**Conclusion:**

In the present study, a correlation was observed between aberrant β-catenin expression and age over 50 years in patients and HER2 negativity, although this association was not statistically significant.

## Introduction

Breast cancer is the most common malignancy in women worldwide and is the second leading cause of cancer death (López-Knowles et al., 2010; Staaf et al., 2010; Wang et al., 2015). Current treatments for breast cancer depend on estrogen receptor(ER), Progesterone receptor (PR), and Human epidermal growth factor receptor2 (HER2) status (López-Knowles et al., 2010; Khan et al., 2018).Knowledge of clinicopathological parameters such as tumor size, tumor grade, and lymph node involvement status is essential in clinical decision making for the patient (Wang et al., 2015).

In addition to the above, identifying novel biomarkers involved in tumor invasion and metastasis can help determine the tumor’s prognosis and new targeted therapies (O’Shaughnessy, 2006; Staaf et al., 2010; Wang et al., 2015). Several signaling pathways, including Wnt, are involved in breast cancer (Khan et al., 2018; Varma et al., 2020). β-catenin is an oncogene (López-Knowles et al., 2010) and is an essential intermediate in the Wnt signaling pathway(Wang et al., 2015). E-cadherin is a molecule involved in cell-cell adhesion in the plasma membrane and helps maintain cell polarity (Guarino et al., 2007; Wang et al., 2015). It has an intracellular region that contains the binding site to interact with catenins (Shen et al., 2016).

Some studies have suggested the role of β-catenin as a proto-oncogene, which causes many cancers, such as colon cancer (Varma et al., 2020).

Recent studies have shown that Wnt/ β-catenin activation and abnormal cytoplasmic/nuclear expression of β-catenin in many human cancers, including breast carcinoma, are associated with increased invasion, metastasis, and poor prognosis (Khramtsov et al., 2010; Shen et al., 2016).

It has also been observed that nuclear and cytoplasmic accumulation of β-catenin, which represents Wnt pathway activation, is more common in basal-like breast cancers, and a significant correlation was found between the aberrant expression of this maker and negativity of hormone receptors and HER2(Khramtsov et al., 2010). 

Due to the lack of sufficient information in this area, particularly in Iran, in the present study, we examined the expression and location of β-catenin in breast cancer specimens and its relationship with clinicopathological parameters.

## Materials and Methods

In this cross-sectional study,88 samples diagnosed as invasive ductal breast carcinoma who underwent mastectomy from 2007 to 2017 were evaluated. The slides and paraffin blocks were retrieved from the archive of the pathology department of Shariati and Sina hospitals, Tehran university of medical science. Tumoral tissue with representative areas (away from necrosis, in situ components, and benign epithelium) were identified and included. The tumor areas selected from the H&E slide were matched to the corresponding block and used for tissue microarray construction. Selected 0.6 mm tissue cores from the donor block were placed in a recipient block, and 5-micron sections were cut from the recipient block and then transferred to a slide. For increasing the accuracy, two cores from each tumor were placed on the slide, and the expression of β-catenin marker through immunohistochemical staining was examined. IHC was performed using Rabbit anti-human β-catenin monoclonal antibody ( clone EP35, Master diagnostica) according to the manufacturer’s protocol. Two pathologists re-examined tumoral tissue from each patient, and different patterns of β-catenin expression were examined. 

Patients’ clinical characteristics, including age, sex, and other information include tumor size, probable lymph node involvement, hormonal profile, and expression of Her2 and Ki67 IHC markers, were investigated. These data were also extracted from medical documents, and the relationship between β-catenin expression and clinicopathological parameters was investigated.

Representative H&E slides were examined and graded according to Nottingham modification of the scarf-Bloom-Richardson (MBR)(Mirzaiian et al., 2020). 

The results related to qualitative variables were calculated and reported as frequency and percentage, and the results about quantitative variables were calculated as average and standard deviation.

Chi-squared test and Fisher’s Exact test were used to evaluate the relationships between qualitative variables. After data collection, the analysis of data was conducted with SPSS version 22.0 software. P-value <0.05 was considered significant.

In this study, the researchers did not intervene, and no cost was imposed on the patient. The results of the survey were reported without mentioning the names and details of the patients. As a result, this study is morally acceptable.

## Results

In total, 88 histologically confirmed invasive breast cancer cases were included in this study and analyzed by IHC for β-catenin expression. Of these, 83(94%) were female, and 5 (6%) were male. The patients’ mean average age was 52 +_13.8 years ( ranged from 27-91 years).

The frequency of different patterns of β-catenin expression is shown in Table 1.

There was no significant correlation between gender and aberrant β-catenin expression.( P-value:0.999). Also, there was no significant correlation between mean age and aberrant β-catenin expression( P.Value: 0.303 and 0.093). However, aberrant expression of β-catenin appears to be more common in people over 50 years of age.

Table 2 shows the age and sex distribution and its correlation with the aberrant β-catenin expression. There was no significant correlation between tumor size and aberrant β-catenin expression (P.Value:0.316). No significant correlation was observed between tumor grade and pattern of β-catenin expression (P.Value:0.213). There was no significant correlation between lymph node metastasis and aberrant β-catenin expression (P.Value: 0.111). There was no significant correlation between ER and PR’s positivity and aberrant β-catenin expression (P.Value: 0.913 and 0.938, respectively). There was a close and significant correlation between HER2 expression and aberrant β-catenin expression( P.Value:0.070). 

The relationship between HER2 and aberrant expression of β-catenin is shown in Table 3. No significant correlation was observed between the expression of Ki67 and aberrant β-catenin expression (P.Value:0.322).

**Figure 1:**
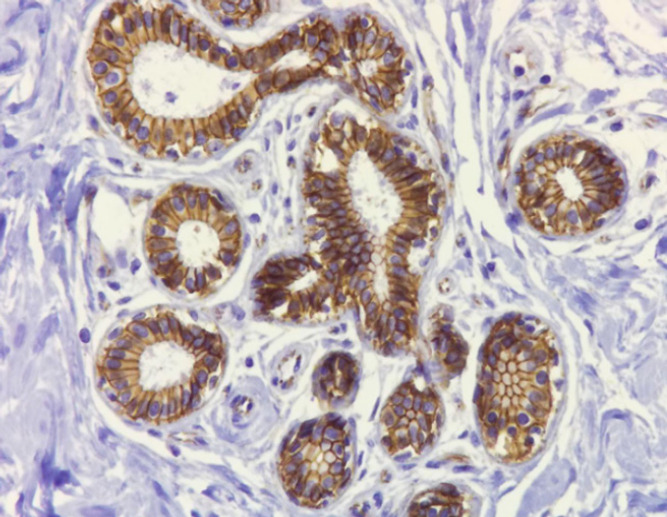
Normal Membranous Expression of β-catenin in Non-Neoplastic Breast Tissue ( IHC 200X).

**Table 1:** The Frequency of Different β-catenin Expression Patterns in Breast Cancer Specimens

β-catenin	Frequency	Percentage
Normal membranous expression	62	70.45
Aberrant (LOM without cytoplasmic and nuclear expression)	12	13.64
Aberrant (LOM with cytoplasmic expression and with/without nuclear expression)	14	15.91
Total	88	100

LOM, Loss of membrane staining

**Table 2:** Patterns of β-catenin Expression and Its Relationship with Age and Sex of the Patient

	Total	Normal	with aberrant expression	P-value
Sex, N (%)				0.999*
Female	83 (94.32)	58 (93.55)	25 (96.15)	
Male	5 (5.68)	4 (6.45)	1 (3.85)	
Age, mean±SD	52.1±13.8	51.1±13.2	54.5±15.2	0.303
≤50	46 (52.27)	36 (58.06)	10 (38.46)	0.093
>50	42 (47.73)	26 (41.94)	16 (61.54)	

*, Using Fisher's exact test

**Figure 2:**
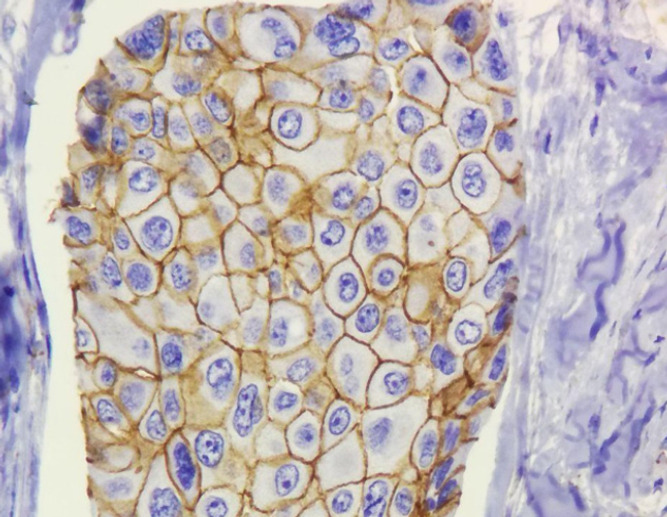
Membranous Expression of β-catenin in Neoplastic Breast Tissue (IHC 400X)

**Table 3:** Association of HER2 and Aberrant Expression of β-catenin

	Total	Normal	with aberrant expression	P-value
HER2, N (%)				0.364*
Negative	55 (62.5)	35 (56.45)	20 (76.92)	
Score 1	12 (13.64)	9 (14.52)	3 (11.54)	
Score 2	8 (9.09)	7 (11.29)	1 (3.85)	
Score 3	13 (14.77)	11 (17.74)	2 (7.69)	
HER2 (dichotomous), N (%)				0.07
Negative	55 (62.5)	35 (56.45)	20 (76.92)	
Positive	33(37.5)	27 (43.55)	6 (23.08)	

*, Using Fisher's exact test

**Figure 3:**
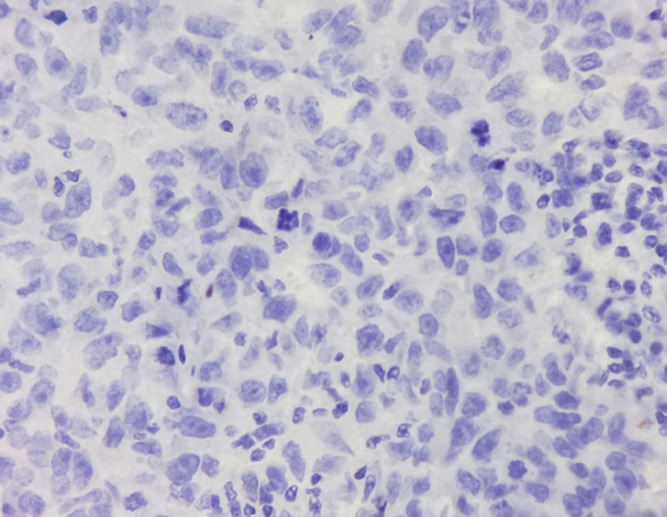
Aberrant Expression of β-catenin without Nuclear and Cytoplasmic Expression (Loss of Membrane Staining, IHC 400X)

**Figure 4:**
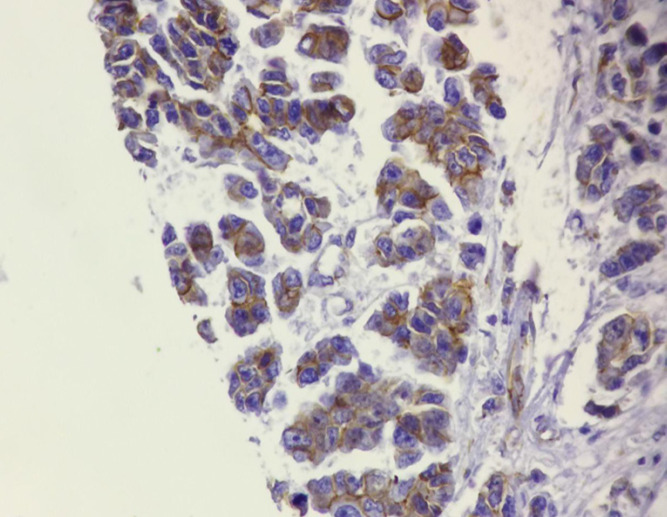
Aberrant Expression of β-catenin (Cytoplasmic Expression, IHC 200X)

## Discussion

β-catenin is involved in carcinogenesis Through the Wnt signaling pathway. This pathway’s activation causes nuclear and cytoplasmic expression of this marker (Tian et al., 2011; Rosa et al., 2015; Varma et al., 2020). Under normal conditions, this marker is expressed in the epithelial cell’s cytoplasmic membrane (Shen et al., 2016).

Aberrant activation of the β-catenin signaling pathway has been observed in many malignancies, including breast cancer. However, its importance in breast cancer is still not well understood.

Contradictory results have been reported regarding the relationship between β-catenin expression and clinicopathological variables. Therefore, there is a need for extensive research in this area (Chung et al., 2004; Varma et al., 2020).

In breast cancer, the type of treatment depends on the hormone receptors, Her2, and the clinical-pathological variables, including tumor size, tumor grade, and lymph node involvement status (López-Knowles et al., 2010; Wang et al., 2015). Therefore, recognizing new biomarkers can be a step towards identifying new treatments for this common cancer.

Previous studies have shown that β-catenin signaling activation in the Wnt pathway and aberrant expression of this marker is associated with tumor progression and metastasis (Rosa et al., 2015). Therefore, familiarity with this biomarker is probably useful in determining patients’ prognosis and survival and recognizing new treatments.

Due to the high prevalence of breast cancer and the lack of sufficient information in this area, particularly in Iran9, in the present study, we examined the expression and location of β-catenin in breast cancer specimens and its relationship with clinicopathological parameters.

In our study, the Tissue microarray (TMA) method was used. In this method, hundreds of tissue samples can be examined simultaneously under the same conditions. Therefore, it saves time, money, and consumption of the reagent. Also, the amount of tissue required in this method is low, and tissues can be preserved for further studies (Mills et al., 1995; Camp et al., 2000; Khouja et al., 2010). After the TMA blocks described in the material and method section were prepared, They underwent immunohistochemical staining for the β-catenin, and different staining patterns of this marker were examined.

In our study, about 70% of the samples showed normal β-catenin membrane expression. In about 30% of the samples, the aberrant expression of this marker was observed. As previously mentioned, aberrant nuclear and cytoplasmic expression of this marker is associated with invasion, metastasis, and poor prognosis (Shen et al., 2016).

In a study, Varma (2020) examined the association of different β-catenin expression patterns with breast carcinoma pathogenesis (Varma et al., 2020). In this study, the expression of β-catenin and Cyclin D1 was evaluated by immunohistochemistry in 82 breast cancer cases. Abnormal β-catenin expression was seen in 80% of breast invasive ductal carcinoma cases.

In the study of wang et al., (2015) The clinical implications of β-catenin protein expression in breast cancer were investigated. In this study, out of 241 patients with breast cancer who underwent radical surgery, abnormal β-catenin expression was observed in 41 patients.

In a study, Lee (2005) examined the prognostic significance of abnormal β-catenin expression in breast carcinoma. In this study, abnormal β-catenin expression was observed in 30 of 55 breast carcinoma cases.

In the study of Shen et al., (2016) The prognostic value of β-catenin and E-cadherin in triple-negative breast cancers was investigated. Most cases of triple-negative breast cancers had an abnormal β-catenin expression.

In a study conducted by Geyer et al., (2011) abnormal β-catenin expression was significantly associated with ER and HER2 negativity. While in the study of Niu et al., (2009) There was a significant relationship between HER2 positivity and abnormal β-catenin expression.

Our study investigates the relationship between HER2 and β-catenin expression in patients. Once the Her2 variable was considered a ranking variable, There was no significant correlation between Her2 expression and β-catenin protein expression using the Chi-square test. Considering Her2 as a two-state variable (negative and positive), this relationship is meaningful at the 10% level.

 In the study of Niu et al., (2009), abnormal β-catenin expression was associated with positive lymph node status and higher histological grade.

In our study, the median Ki67 percentage was 15%. Tumors were divided into two groups based on the Ki67 rate ( ≤15% and >15%). In our study, there was no significant relationship between Ki67 expression and abnormal β-catenin expression. Also, there was no meaningful relationship between patient sex, histological tumor grade, and lymph node metastasis with abnormal β-catenin expression.

In the study of Shen et al., (2016) Tumor size, pathologic tumor stage, lymph node involvement, and decreased β-catenin membrane expression were associated with poor prognosis and decreased patient survival.

In our study, the tumor’s median size was 4 cm, and the tumors were divided into two groups in terms of size (≤ 4cm and >4cm). There was no significant relationship between tumor size and abnormal expression of β-catenin.

In a study by Lee (2005) abnormal β-catenin expression was significantly associated with lymph node metastasis and poor prognosis in patients.

In a Study by Khramtsov et al., (2010) abnormal nuclear and cytoplasmic β-catenin expression was higher in basal-like breast cancers than in others. Also, in this study, the survival rate in patients was evaluated, Which was found in cases where there was an abnormal expression of β-catenin, survival was lower than β-catenin membranous expression.

In the study of Khan et al., (2018) deregulation of the Wnt pathway was not associated with the age of onset of the disease, the tumor grade, and triple-negative breast cancers.

Our study observed a correlation between aberrant β-catenin expression and age over 50 years in patients, although this association was not statistically significant.

In a study by Lee (2005) β-catenin lack of membrane staining was associated with a higher grade of tumor and negative ER. There was no significant relationship between ER and PR positivity and the β-catenin’s abnormal expression in our study. 

One of the critical limitations of our study is the lack of clinical outcomes in patients. This limitation was due to the lack of access to patients’ contact information. Besides, information about the patients’ clinical-stage was not available.

In conclusion, based on previous studies’ results, an aberrant β-catenin expression is associated with the tumor’s aggressive behavior, metastasis, and poor prognosis in breast carcinomas. As discussed, there are conflicting results in various studies regarding aberrant β-catenin expression with clinicopathological parameters. 

In our study, a correlation was observed between aberrant β-catenin expression and age over 50 years in patients and HER2 negativity, although this association was not statistically significant.

What is clear from the results of some studies is that β-catenin can be used as a prognostic marker and predictor of survival in patients. Therefore, familiarity and use of this marker can help determine patients’ survival and discover new breast cancer treatments. We propose further studies with larger sample sizes and information from patient follow-up.

## Author Contribution Statement

None.
